# A combined electrohydrodynamic atomization method for preparing nanofiber/microparticle hybrid medicines

**DOI:** 10.3389/fbioe.2023.1308004

**Published:** 2023-11-15

**Authors:** Liang Sun, Jianfeng Zhou, Yaoning Chen, Deng-Guang Yu, Ping Liu

**Affiliations:** ^1^ Department of Urology, Shandong Provincial Hospital Affiliated to Shandong First Medical University, Jinan, China; ^2^ School of Materials and Chemistry, University of Shanghai for Science and Technology, Shanghai, China; ^3^ The Base of Achievement Transformation, Shidong Hospital Affiliated to University of Shanghai for Science and Technology, Shanghai, China

**Keywords:** coaxial electrospraying, electrospinning, micro/nano hybrids, sequential release, prostatitis

## Abstract

Bacterial prostatitis is a challenging condition to treat with traditional dosage forms. Physicians often prescribe a variety of dosage forms with different administration methods, which fail to provide an efficient and convenient mode of drug delivery. The aim of this work was to develop a new type of hybrid material incorporating both electrosprayed core-shell microparticles and electrospun nanofibers. A traditional Chinese medicine (Ningmitai, NMT) and a Western medicine (ciprofloxacin, CIP) were co-encapsulated within this material and were designed to be released in a separately controlled manner. Utilizing polyvinylpyrrolidone (PVP) as a hydrophilic filament-forming polymer and pH-sensitive Eudragit^®^ S100 (ES100) as the particulate polymeric matrix, a combined electrohydrodynamic atomization (EHDA) method comprising coaxial electrospraying and blending electrospinning, was used to create the hybrids in a single-step and straightforward manner. A series of characterization methods were conducted to analyze both the working process and its final products. Scanning electron microscopy and transmission electron microscopy revealed that the EHDA hybrids comprised of both CIP-PVP nanofibers and NMT-ES100 core-shell microparticles. Multiple methods confirmed the rapid release of CIP and the sustained release of NMT. The antibacterial experiments indicated that the hybrids exhibited a more potent antibacterial effect against *Escherichia coli dh5α* and *Bacillus subtilis Wb800* than either the separate nanofibers or microparticles. The amalgamation of fibrous nanomedicine and particulate micromedicine can expand the horizon of new types of medicines. The integration of electrospinning and coaxial electrospraying provides a straightforward approach to fabrication. By combining hydrophilic soluble polymers and pH-sensitive polymers in the hybrids, we can ensure the separate sequential controlled release of CIP and NMT for a potential synergistic and convenient therapy for bacterial prostatitis.

## 1 Introduction

Nanomedicines, an emerging trend in healthcare, combine traditional medicines, pathology, material science, and advanced materials conversion methods to bring about effective treatments (Yap et al., 2021; Cai et al., 2022; 2023; Meng et al., 2022; Tang et al., 2022; Wu et al., 2022). Over the past 2 decades, medicated nanofibers, a branch of nanomedicines, have rapidly grown, and their potential applications cover various diseases (Chen et al., 2022; Shen et al., 2022; Xie et al., 2022; Lang et al., 2023; Qian et al., 2023; Tan et al., 2023). However, to our knowledge, no studies have explored the treatment of prostatitis through electrospun nanofibers to date.

The prostate, a deep-seated organ in the adult male pelvic cavity (Murugesan and Raman, 2022), presents challenges for direct treatment methods, such as infusion, when inflamed (Bertelli et al., 2022; Jiang et al., 2022; Rubegeta et al., 2023). Consequently, prostatitis is often challenging to treat fully. Over the years, various synthetic chemical molecules (such as ciprofloxacin (CIP), norfloxacin, flomoxef, and cilastatin) (Dan et al., 1987; Schaeffer and Darras, 1990; Kuiper et al., 2020; Nakamura et al., 2020; Zheng J. et al., 2022) and herbal medicines have been developed into oral dosage forms (Hu et al., 2022; Xu et al., 2022; Gao et al., 2023; Zhong et al., 2023). In clinics, doctors often combine traditional Chinese medicines with Western medicine and antibiotics for improved therapeutic effects on patients with chronic bacterial prostatitis. However, the complexity of medication methods can cause inconvenience for patients (Alzahrani et al., 2022; Bouiller et al., 2022; Kuiper et al., 2022; Marino et al., 2022). We propose that these medicines could be integrated into a single medical material, improving drug delivery effectiveness and functional performance through controlled release profiles.

Electrospinning and electrospraying are two forms of electrohydrodynamic atomization (EHDA) methods (He et al., 2022; Ji et al., 2023; Xu et al., 2023; Yang et al., 2023; Yu and Xu, 2023). The terminology derives from the material conversion mechanism, in which electrostatic energy (electro) is utilized to process working liquid (hydro) through a powerful interactive process (dynamic). During this process, the atomization of the liquids transfers the fluid into solid products (Bai et al., 2022; Cao et al., 2022; He et al., 2022; Song et al., 2022; Wang H. et al., 2023; Chen X. et al., 2023; Du et al., 2023; Ji et al., 2023; Xu et al., 2023; Yang et al., 2023; Yao et al., 2023; Yu and Xu, 2023). For electrospinning and electrospraying, nanofibers and microparticles are the main products, respectively (Zhou et al., 2023c; 2023a). These products have demonstrated significant potential in clinical applications for treating a range of diseases (Yao et al., 2018; Brimo et al., 2022; Wang et al., 2022), and their related drug delivery applications extend from common wound dressings (Yang et al., 2020; Jaberifard et al., 2023) to transmembrane, transdermal and oral administration, implants, and even injections (after the self-emulsification of the electrospun nanofibers) (Pérez-González et al., 2019; Hou et al., 2023).

After 3 decades of development, both electrospinning and electrospraying have evolved into various subbranches. The most common single-fluid electrospinning differentiates into 2-fluid coaxial (Xing et al., 2018; Shen et al., 2020; Huang et al., 2022; Li et al., 2022; Yao et al., 2022) and side-by-side processes (Lv et al., 2023a; 2023b), 3-fluid triaxial (Wang et al., 2020; Wang et al., 2023 M.; Guler et al., 2023), tri-layer side-by-side (Liu et al., 2022; Feng et al., 2023), and the combination of coaxial and side-by-side processes (Song et al., 2023a). Consequently, a range of complex electrospun fibrous structures have been successfully fabricated for extraordinary applications (Han et al., 2022; Tabakoglu et al., 2023). Simultaneously, electrospraying has also shifted to multifluid processes for producing functional particulate structures (Zheng S. et al., 2022; Li et al., 2023). However, there have been limited reports exploring the combination of electrospinning and electrospraying, and the corresponding amalgamation of electrospun nanofibers and electrosprayed particles.

In light of the aforementioned development status of EHDA and prostatitis treatments, this study investigated the combination of coaxial electrospraying and single-fluid electrospinning. Through this approach, we successfully fabricated a novel type of hybrid material composed of electrosprayed core-shell microparticles and electrospun nanofibers in a single-step and straightforward manner. The hybrids were designed to leverage the advantages of electrospun nanofibers (such as small diameter, large surface area, and high porosity) and the excellent solubility of the hydrophilic polymer polyvinylpyrrolidone (PVP) (Uhljar et al., 2021; Shi et al., 2023) for a pulsatile release of the loaded CIP. This release mechanism has the potential to quickly alleviate patient pain and discomfort. Simultaneously, the hybrids were designed to utilize the electrosprayed core-shell microparticles and the pH-sensitive polymeric excipients Eudragit^®^ S100 (ES100) (Miranda-Calderon et al., 2022; Yu et al., 2022; Abdi et al., 2023; Ao et al., 2023) to provide a colon-targeted sustained release profile of a commercial herbal medicine for continuous therapeutic effects. The separately controlled sequential release of two types of medicines demonstrated synergistic antibacterial activity, suggesting promise for potential clinical applications in treating chronic and acute bacterial prostatitis.

## 2 Materials and methods

### 2.1 Materials

The filament-forming soluble polymer PVP K60 (molecular weight 360,000) was sourced from BASF Co., Ltd. (Shanghai, China). ES100 (average molecular weight approximately 135,000) was provided by Rohm GmbH & Co. KG (Darmstadt, Germany). CIP was procured from China National Pharmaceutical Group Corporation (Shanghai, China). Ningmitai (NMT) (Approval number of China Food and Drug Administration: 20025442) was purchased from Lao-Bai-Xing Big Pharmacy (Shanghai, China). Anhydrous ethanol, sodium hydroxide, and hydrochloric acid were purchased from Sinopharm Chemical Reagent Co., Ltd. (Shanghai, China). All water used was double distilled. All other chemicals and reagents were of analytical grade.

### 2.2 The EHDA processes for the fabrications

The concentric spraying head and the entire EHDA apparatus were custom-made for this study. Three fluid drivers (KDS100 or KDS200, Kole-parmer, United States) were employed to accurately deliver the working fluids to the electrical fields. Two separate high voltage generators (2000 ZGF/6 mA, Wuhan Huatian Co., Ltd., Wuhan, China) supplied the electrostatic energy to initiate and maintain the electrospinning and coaxial electrospraying processes. A custom-made rotating (axial fixed) plate was used for uniform collection of nanofibers and core-shell microparticles into a homogeneous hybrid film. Table 1 includes the working fluids and operational parameters for creating Sample 1 (S1, electrospun polyvinylpyrrolidone-ciprofloxacin (PVP-CIP) nanofibers), Sample 2 (S2, electrosprayed ES-NMT core-shell microparticles), and Sample 3 (S3, EHDA hybrids composed of both PVP-CIP nanofibers and ES-NMT core-shell microparticles), determined through pre-experiments.

**TABLE 1:** The fabrication parameters optimized in the EHDA processes.

No.	Working process		Working fluid/flow rate	Conditions^d^		Drug contents	Morphology
				*V* (kV)	*D* (cm)		
S1	Electrospinning		^a^ Fluid 1/1.0 mL/h	0.8	15	12.5% CIP	Nanofibers
S2	Coaxial electrospraying		^b^ Fluid 2/0.4 mL/h;^c^ Fluid 3/1.0 mL/h	18	20	29.4% NMT	Micro-particles
S3	Combined EHDA	Electrospinning	Fluid 1/1.0 mL/h	0.8	15	6.8% CIP; 13.5% NMT	Hybrids
		Coaxial electrospraying	Fluid 2/0.4 mL/h; Fluid 3/1.0 mL/h	18	20		

^a^ Fluid 1: A total of 1.0 g CIP, and 7.0 g PVP, were co-dissolved in 100 mL anhydrous ethanol.

^b^ Fluid 2: An amount of 2.0 g ES100 was dissolved into 100 mL anhydrous ethanol as the shell working fluid.

^c^ Fluid 3: An amount of 2.0 g NMT, powders and 4.0 g ES100 were co-dissolved into 100 mL 75% (v/v) ethanol aqueous solution.

^d^ *V* and *D* represent the applied voltage (kV) and the working distance between the nozzle of spinneret and the collector, respectively.

### 2.3 Characterization

#### 2.3.1 Morphology and inner structure

A scanning electron microscope (FEI Quanta G450 FEG, Inc., Hillsboro, OR, United States) was used to evaluate the surface morphologies of the EHDA products (S1, S2, and S3). Before evaluation, samples were gold sputter-coated under argon to render them electrically conductive, and images were taken at an excitation voltage of 10 keV. The inner structures were assessed using a transmission electron microscope (TEM, JEM2100F, JEOL, Tokyo, Japan). The samples were prepared by fixing a lacy carbon-coated copper grid on the rotation collector for approximately 2 min.

#### 2.3.2 Physical state and compatibility

X-ray diffraction (XRD) analysis was conducted on a Bruker D8 ADVANCE diffractometer (Bruker, Bremen, Germany). The XRD pattern was recorded from 10° to 60° in continuous mode with a step size of 0.02° and a scanning speed of 5°/min. ATR-FTIR spectra were recorded by a Spectrum 100 spectrometer (Perkin-Elmer, Waltham, MA, United States). The scanning range was from 500 cm^-1^ to 4,000 cm^−1^ with a resolution of 2 cm^−1^.

### 2.4 Drug release profiles

#### 2.4.1 Homemade methods for assessing the fast release of CIP

The rapid dissolution of nanofibers in the S3 hybrids was assessed using two homemade methods. One approach is the artificial tongue method, where a circular sheet of the S3 hybrids was placed on the surface of a damp piece of paper. The alternative method involved dripping a drop of water onto the hybrids, which were collected on a glass slide. All procedures were recorded using a digital camera (Canon PowerShot SX50HS, Tokyo, Japan).

#### 2.4.2 *In vitro* dissolution tests and quantitative measurements of CIP and NMT release from the EHDA products

*In vitro* dissolution tests were conducted in accordance with the Chinese Pharmacopoeia (2020 Ed.). The paddle method was performed using an RCZ-8A dissolution apparatus (Tianjin University Radio Factory, China) with seven vessels. The test conditions involved a rotation speed of 50 rpm and a dissolution media temperature of 37°C ± 1°C. For the electrospun nanofiber S1, the dissolution medium (600 mL) was 0.01 N HCl (pH = 2.0). For the electrosprayed microparticles S2, the dissolution medium (600 mL) was 0.01 N HCl (pH = 2.0) for the first 2 h to imitate the artificial gastric juice, and subsequently, an equivalent volume of sodium hydroxide was added to the dissolution medium to adjust the pH value to 7.0, simulating artificial intestinal fluid. For the EHDA hybrids S3, the dissolution medium (600 mL) was 0.01 N HCl (pH = 2.0) for 2 min. Later, the microparticles in the S3 hybrids were obtained via centrifugal treatments and redispersed into 600 mL of fresh pH 2.0 HCL. This step was performed to eliminate the possible influence of dissolved CIP on the quantitative measurements of NMT release from the microparticles for 118 min. Finally, an equivalent volume of sodium hydroxide was added to the dissolution medium to adjust the pH value to 7.0, again imitating the artificial intestinal fluid.

At predetermined time intervals, a 5.0 mL volume of dissolution media was withdrawn for sampling, and an equal volume of fresh media was added to maintain a constant volume. The absorbance of the samples was measured using a UV‒vis spectrophotometer (Unico Instrument Co., Ltd., Shanghai, China). The amount of CIP and NMT present in the samples could then be calculated using their calibration curves.

### 2.5 Antibacterial performances

The antibacterial efficacy of the EHDA products S1, S2, and S3, along with the raw NMT powders, was evaluated using the plate count method. *Escherichia coli* dh5α (*Escherichia coli* dh5α) and *Bacillus subtilis* (Wb800) were selected as representative Gram-negative and Gram-positive microorganisms, respectively. The procedure, based on previous literature (Zhou et al., 2023b), is outlined as follows:

1) 5.0 mL of sterilized Luria–Bertani (LB) broth was added to an Erlenmeyer flask.
2) 50.0 mg of the EHDA products were introduced to the LB broth, which contained approximately 1.5×10^5^ colony-forming units (CFU) of both *E. coli* dh5α and Wb800.
3) The mixtures were cultured in a shaking incubator for 12 h at 37°C ± 1°C.
4) A volume of 100 μL of each cell solution was seeded onto LB agar using a surface spread plate method.
5) The plates were incubated at 37°C for 8 h. The numbers of CFUs were subsequently counted.

The blank control for comparison was pure phosphate-buffered saline, and the third and fifth steps were repeated for this control. The antibacterial efficacy (ABE, %) of the samples was calculated using the following equation:

$$ABE\;\left(\%\right)=\left(Np\;–\;Nt\right)/Np\times 100\%$$

where Np and Nt represent the numbers of viable bacterial colonies in the blank control (with pure phosphate-buffered saline buffer added) and the tested samples, respectively. All experiments were performed in triplicate.

## 3 Results and discussion

### 3.1 The combination of a coaxial electrospraying and a blending electrospinning

**FIGURE 1:**
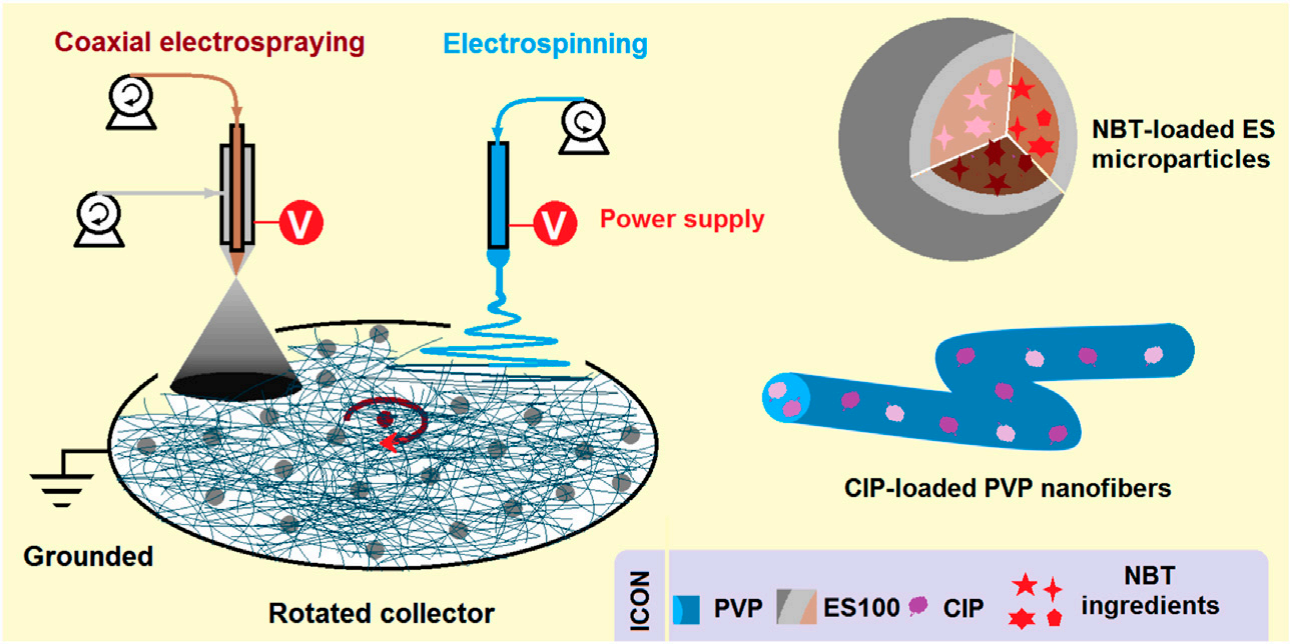
A schematic showing a new EHDA process, which is a combination of coaxial electrospraying and single-fluid blended electrospinning for fabricating hybrids containing both monolithic CIP-PVP fibrous nanocomposites and core-shell ES-NMT microparticles with a blank shell ES100 coating.

The combined EHDA method consists of coaxial electrospraying and single-fluid blending electrospinning, as shown in Figure 1. As with the traditional EHDA apparatus, this system has four main elements: spinnerets to guide the working fluids into the electrical fields, pumps to quantitatively deliver the working fluids, power supplies to provide high voltages, and a collector for the deposition of hybrids. The differences lie in the following aspects:

1) Two different spinnerets are needed: a concentric one for coaxial electrospraying, and a stainless steel capillary for single-fluid blending electrospinning.
2) Two power supplies are required to provide varying high voltage values for the simultaneous implementation of electrospraying and electrospinning.
3) For uniform collection of nanofibers and core-shell particles, the collector should be rotated during the collection process.

The resulting core-shell particles are primarily on the microscale. The shell sections are composed of the blank, pH-sensitive polymer ES100, while the core sections consist of a composite of ES100 and the Chinese herbal medicine NMT. These arrangements should benefit the sustained release of the herbal medicine in the colon region after oral administration. The PVP-drug nanofibers, produced from the electrospinning process, are homogenous polymer-based nanocomposites, as demonstrated in previous studies (Uhljar et al., 2021).

**FIGURE 2:**
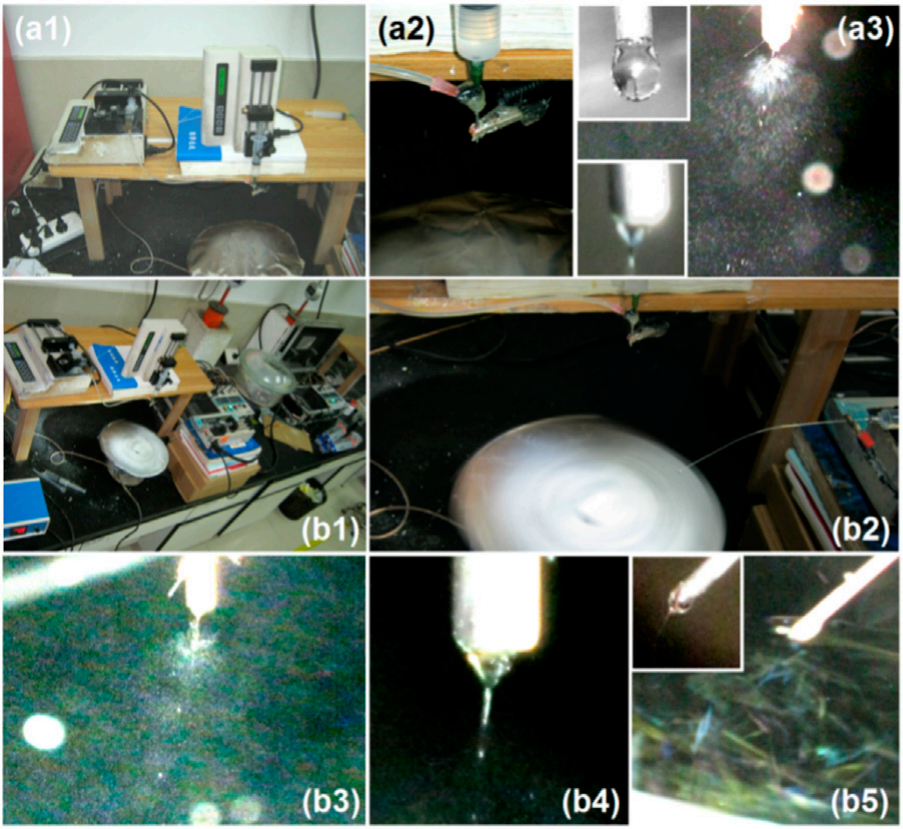
Digital observations of the EHDA working processes. **(a1–a3)** The coaxial electrospraying processes for creating the microparticles S2: **(a1)** an overview of the coaxial electrospraying apparatus; **(a2)** the convergence of two working fluids and high power supply on the spinneret; **(a3)** a typical electrospraying process, the upleft and downleft insets show the non-charged core-shell droplet and charged compound Taylor cone, respectively. **(b1–b5)** The combined EHDA process comprising both coaxial electrospraying and blended electrospinning: **(b1)** an overview of the apparatus for implementing the combined EHDA processes; **(b2)** the fluids and energy transportation to the electric field and the collections of hybrids; **(b3)** the simultaneous coaxial electrospraying; **(b4)** the compound Taylor cone initiating the coaxial spraying; **(b5)** the simultaneous single-fluid blending electrospinning, the upper left inset shows a typical Taylor cone.

The coaxial electrospraying process observations are shown in Figures 2a1–2a3. In Figure 2a1, the homemade electrospraying system consists of two syringe pumps, a collector, a power supply, and a concentric spinneret. The connections of the concentric spinneret with the two working fluids and power supply are evident in Figure 2a2. The syringe containing the shell ES solution is directly inserted into the concentric spinneret downward, securing the spinneret in the apparatus. The core ES100-NMT solution is driven to the spinneret via a highly elastic silicone tube. An alligator clip is used to transfer the electrostatic energy to the working fluids. In Figure 2a3, a typical electrospraying process is captured, involving three typical stages: Taylor cone formation, straight fluid jet, and Coulomb explosion. Before the application of high voltage, the shell and core fluids form a core-shell round droplet under the nozzle of the spinneret (the top-left inset of Figure 2a3). After the high voltage is applied, reaching 18 kV, a stable Taylor cone is formed, as shown by the bottom-left inset of Figure 2a3, ensuring a continuous and robust spraying process.

The digital images showcasing the combined EHDA processes are shown in Figures 2b1–2b5. An overview of the EHDA apparatus used to generate the hybrids is given in Figure 2b1, which includes three pumps, two high voltage generators, and an axially fixed rotating collector. The simultaneous operation of electrospinning and coaxial electrospraying is displayed in Figure 2b2. Here, the two spinnerets are separated by a horizontal distance of 18 cm and a vertical distance of 5 cm. At these distances, the two electric fields have minimal influence on each other, as confirmed by the images in Figure 2b3 (the spraying process), Figure 2b4 (the compound Taylor cone and the straight fluid jet), and Figure 2b5 (the blended electrospinning). In these images, the straight fluid jets continue the working processes vertically, with few deviations due to charge repulsion. The top-left inset in Figure 2b5 shows an angled Taylor cone formed by the synergistic actions of electrical forces, the surface tension of the PVP-CIP working fluid, and the fluid’s gravity.

### 3.2 The SEM and TEM images of the EHDA hybrids

**FIGURE 3:**
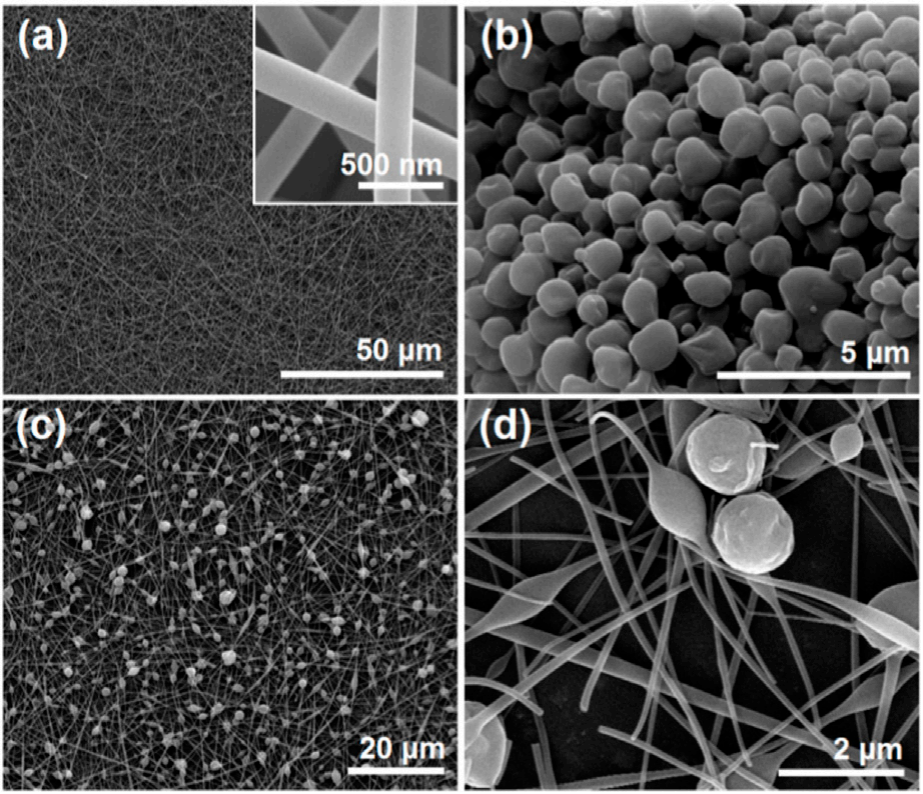
SEM images of EHDA products from different working processes: **(A)** nanofibers S1 from the blended electrospinning process; the upper right inset shows an enlarged SEM image; **(B)** microparticles S2 from the coaxial electrospraying process; **(C,D)** the hybrids containing both microparticles and nanofibers under different magnifications.

The scanning electron microscopy (SEM) images of three EHDA products from different working processes are presented in Figure 3. Figure 3A shows the electrospun PVP-CIP nanofibers (S1) with a fine linear morphology. The upper-right inset of Figure 3A illustrates that these nanofibers have a smooth surface, devoid of discernible particles. The electrosprayed microparticles (S2) appear round with few satellites, likely due to the easy splitting of the dilute shell ES solution. Both Figures 3C, D display SEM images of the EHDA hybrids at varying magnifications. The hybrids are evidently comprised of both electrospun nanofibers and electrosprayed core-shell microparticles. However, the nanofibers demonstrate a less uniform diameter distribution than S1 and exhibit concessional spindles, likely resulting from the influence of the dual electrical fields. Although the choice of filament-forming polymers for electrospinning is limited, there are no constraints on developing polymer-based functional nanofibers (Wang et al., 2017; Song et al., 2023b; Wang and Feng, 2023; Yu and Zhou, 2023) The current creation of hybrids—with a combination of outer shape, inner structure, multiple components, and their designed spatial distribution—represents a successful manifestation of this approach.

**FIGURE 4:**
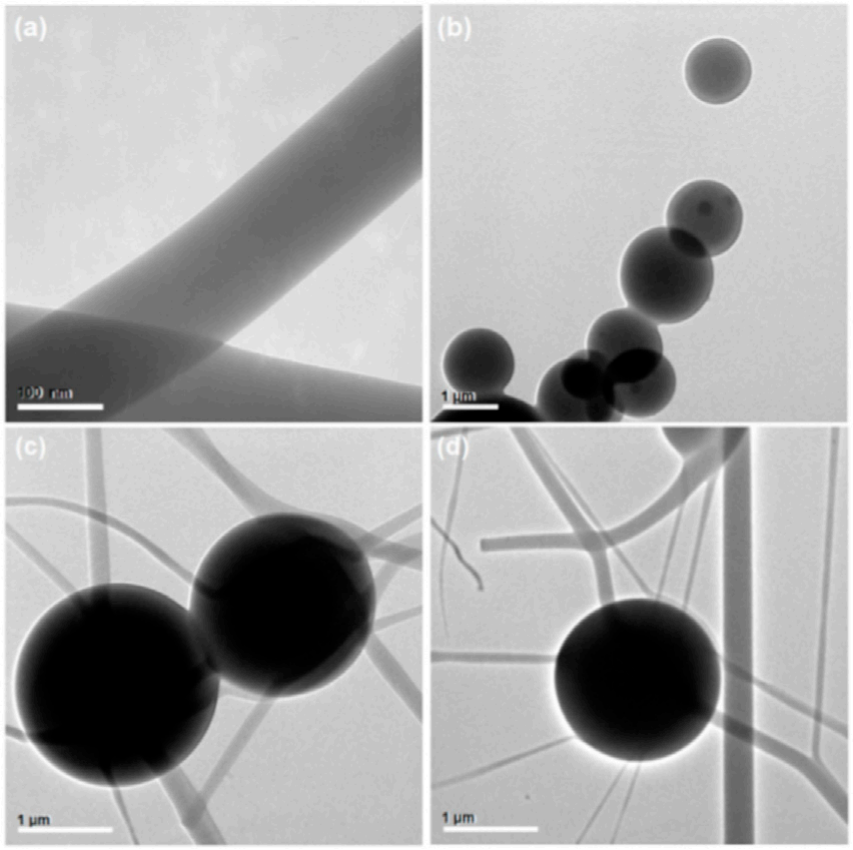
TEM images of EHDA products from different working processes: **(A)** nanofibers S1 from the blended electrospinning process; **(B)** microparticles S2 from the coaxial electrospraying process; **(C,D)** hybrids containing both microparticles and nanofibers.

The TEM images of three EHDA products from different working processes are presented in Figure 4. Figure 4A shows the electrospun PVP-CIP nanofibers (S1) as homogeneous entities with a uniform gray level, implying no discernible phase separation. The electrosprayed microparticles (S2) display clear core-shell structures; the core sections exhibit a deeper gray level than the shell sections. Darker particles sporadically appear in the core sections of microparticles S2, likely due to re-crystallization of NMT components. Figures 4C, D present images of the EHDA hybrids, indicating that the hybrids are a composite of electrospun nanofibers and electrosprayed core-shell microparticles.

### 3.3 The physical state of the active ingredients and their compatibility

**FIGURE 5:**
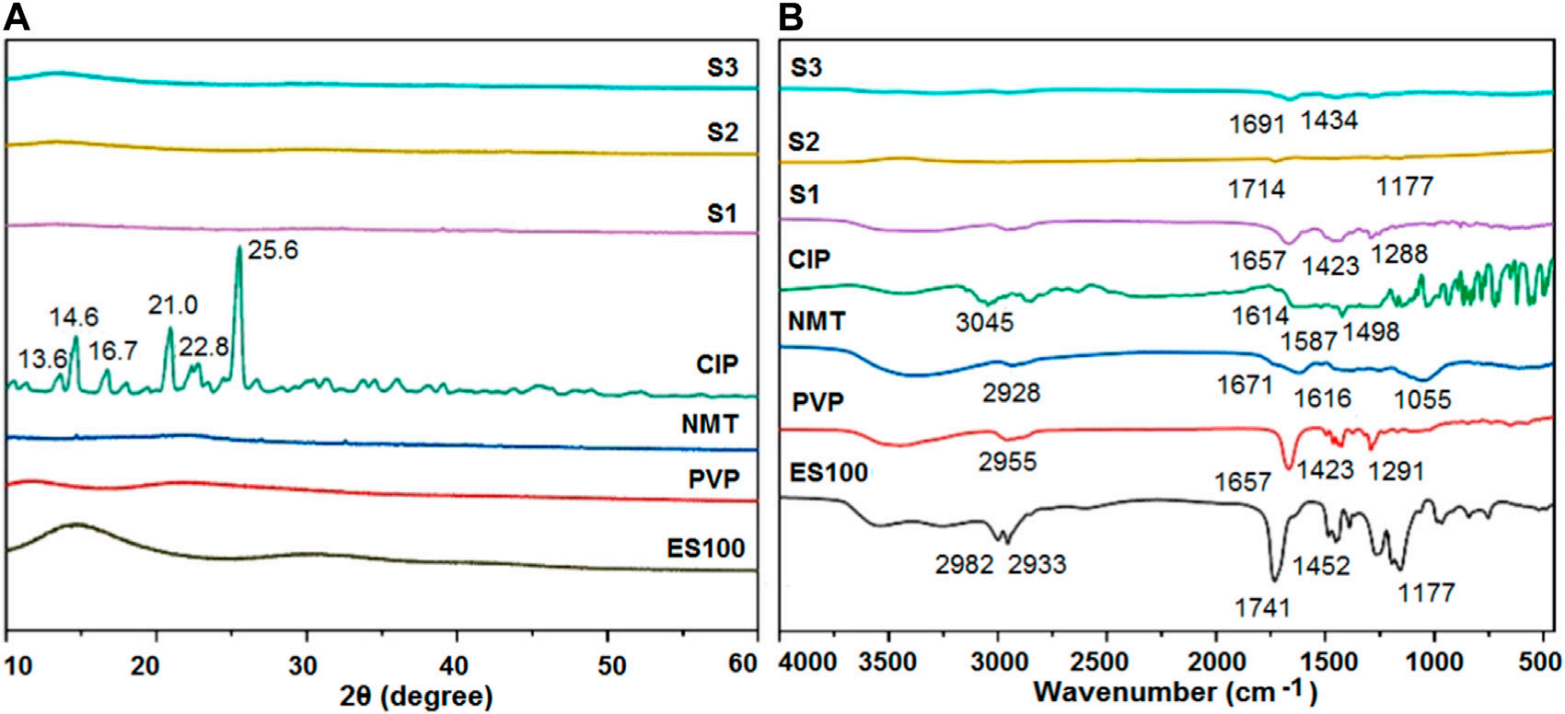
Compatibility and physical state of the raw materials (ES100, PVP, NMT, and CIP) and their EHDA products S1, S2 and S3 from different processes: **(A)** XRD; and **(B)** ATR-FTIR.

XRD characterization analyses were conducted on the raw materials, particles, and fibers, as shown in Figure 5A. The XRD spectra of ES100 and PVP show broad peaks in the range of 10°–35°, indicating their amorphous polymeric nature. The XRD curve of the Chinese herbal medicine features several sharp peaks, suggesting the presence of crystalline ingredients in raw NMT. As expected, characteristic Bragg peaks were observed in the XRD curve of the crystalline drug CIP, with peak positions at 13.6°, 14.6°, 16.7°, 21.0°, 22.8°, and 25.6°. Interestingly, when CIP was co-electrospun with PVP, these characteristic peaks vanished in the PVP-CIP curve of nanofibers (S1). Furthermore, microparticles S2, obtained from the coaxial electrospraying of Chinese herb medicine NMT and ES100, exhibited no characteristic NMT peaks, indicating that all the crystalline drugs were transformed into an amorphous state within the polymeric matrices. The XRD patterns of the hybrids also appeared amorphous.

To further explore the compatibility and interactions among the materials, ATR-FTIR measurements were performed on the raw materials (ES100, PVP, NMT, and CIP) and their respective EHDA products S1, S2, and S3 from different processes. As shown in Figure 5B, the characteristic peaks in the CIP spectra—1,614, 1,587, and 1,498 cm^−1^—can be attributed to the vibrations of benzene rings. The NMT spectra displayed several characteristic peaks at 1,671, 1,616, and 1,055 cm^−1^, reflecting the presence of -C=O groups in their molecules. However, the FTIR spectra of nanofibers (S1) and microparticles (S2)—prepared through electrospinning or electrospraying CIP and NMT with PVP and ES100, respectively—did not show the corresponding drug characteristic peaks. These observations suggest extensive secondary interactions between the drug molecules and polymer matrices. The S3 spectra showed two characteristic peaks at 1,691 cm^-1^ and 1,434 cm^−1^, likely representing compound peaks from the absorbance of both PVP-CIP and ES100-NMT—a testament to the new types of hybrids containing both microparticles and nanofibers.

### 3.4 *In vitro* drug release profiles

**FIGURE 6:**
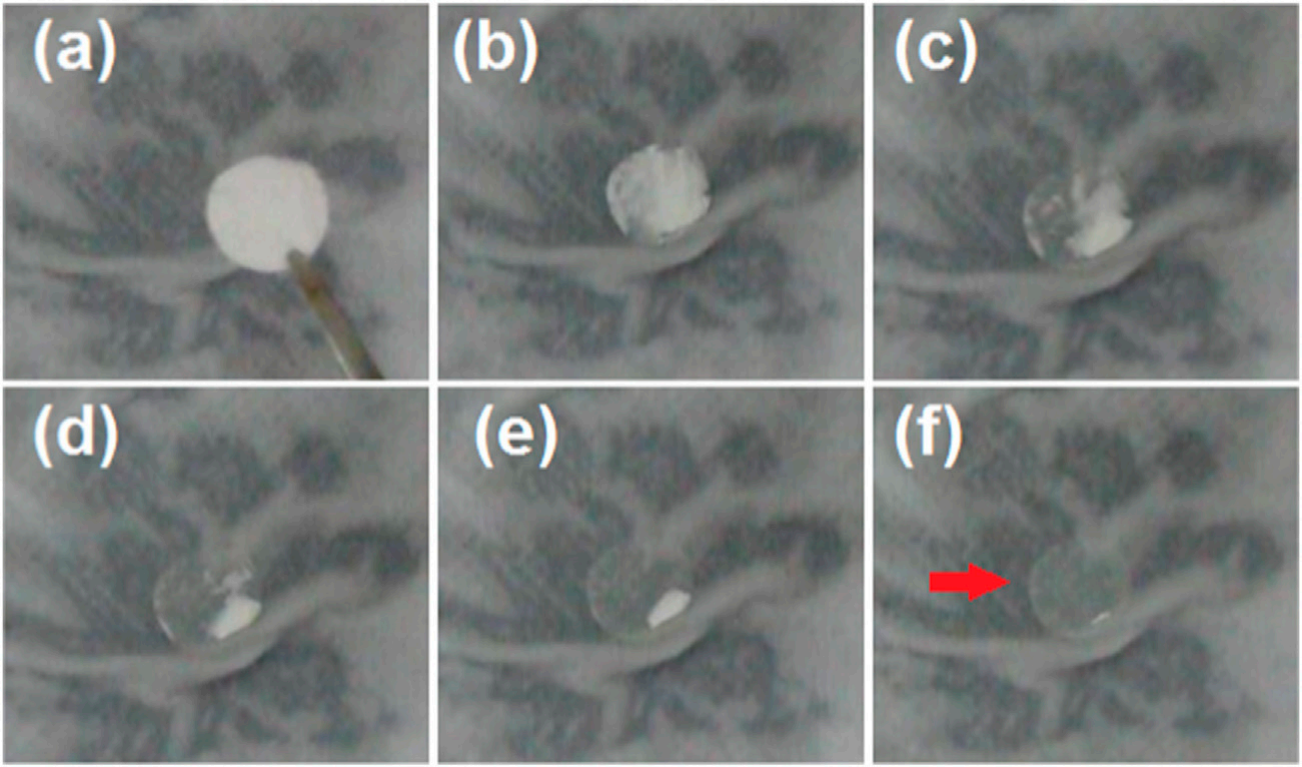
An artificial tongue (a layer of wet paper) was exploited to exhibit the fast dissolution of the nanofibers. The time cost of the sequential dissolution processes from **(A–F)** is 21 s, and the red arrow in **(F)** indicates the residue circle due to the microparticles.

To verify the rapid dissolution of the PVP-CIP nanofibers within the hybrids, an artificial tongue was simply prepared using wet paper. A sheet of hybrids with a circular diameter of 10 cm was placed on wet paper, and the processes were recorded with a camera. The successive results are depicted in Figure 6, with the entire sequential dissolution process from (a) to (f) taking 21 s. After dissolution, an indistinct mark remains on the wet paper (as indicated by the red arrow in Figure 6F), likely due to the microparticles loaded in the hybrids, since PVP becomes transparent after dissolution.

**FIGURE 7:**
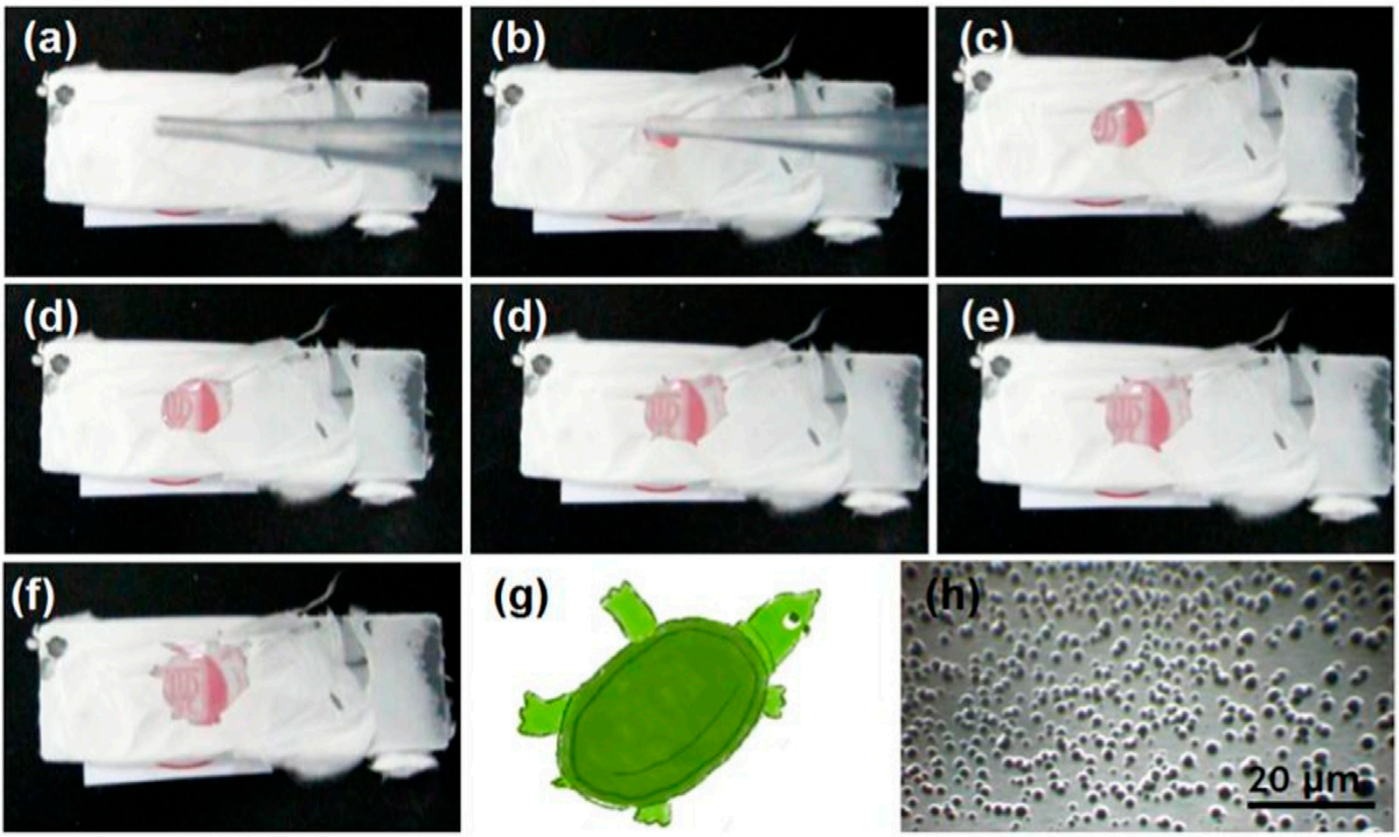
The phenomena when a drop of water was dripped on the collected fibrous films, the time cost from **(A–F)** is 7 s; **(G)** is a diagram of turtle similar to the shape in **(F)**; and **(H)** is the optical image of microparticles left in the center of turtle in **(F)**.

To further elucidate the rapid dissolution of the PVP-CIP nanofibers in the hybrids, a glass slide was used to collect some hybrids, as shown in Figure 7A. After a drop of water was placed on the collected hybrids S3, a dissolution circle rapidly expanded, as indicated from Figures 7B–F, taking approximately 7 s. Although the dissolved PVP-CIP should be transparent, the red circle appeared somewhat obscure, likely due to the loaded microparticles. Interestingly, the circle did not expand uniformly, forming a shape akin to a soft-shelled turtle, as illustrated in Figure 7G. The microparticles in hybrids S3 likely caused this phenomenon. When examined under an optical microscope, many particles could be discerned at the center of the “turtle,” as shown in Figure 7H.

**FIGURE 8:**
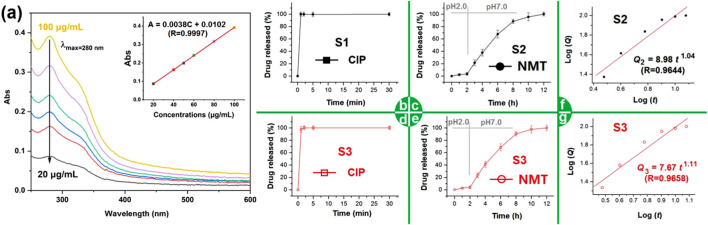
The *in vitro* dissolution profiles of the different EHDA products: **(A)** the scanning curves of NMT solutions from 250 to 600 nm, the upright inset shows the achieved NMT standard line; **(B)** the CIP release profile from nanofibers S1; **(C)** the NMT release profiles from nanofibers S2; **(D,E)** the CIP and NMT release profiles from hybrids S3, respectively; **(F)** and **(G)** the drug NMT sustained release mechanisms from electrosprayed S2 and the EHDA hybrids S3, respectively.

NMT contains several active ingredients, and the UV-scanning curves of different concentrations are included in Figure 8A. Based on the absorbance at various concentrations, a linear standard equation can be constructed for quantitative analysis of the NMT released from both microparticles S2 and the hybrids S3. The equation is $A=0.0038\;C+0.0102\left(R=0.9997\right)$ within the range from 20 to 100 μg/mL, where A represents the absorbance and C represents the concentration of NMT in the tested samples. Concurrently, the *in vitro* rapid release profiles of CIP from PVP-CIP nanofibers S1 and hybrids S3 were also quantitatively detected using UV‒vis spectroscopy.

Fast dissolution of poorly water-soluble drugs is common for many active ingredients in traditional dosage forms (Ejeta et al., 2022; Köse et al., 2022; Assi et al., 2023; Yu and Huang, 2023). Figure 8B displays the CIP release profile from S1. As anticipated, the CIP-PVP nanofibers S1 released the loaded CIP instantaneously. Within 1 minute, the nanofibers S1 completely dissolved, releasing the encapsulated CIP into the dissolution medium. This result arises from several factors, including the solubility of PVP, the extensive surface area and small diameter of nanofibers, the porosity of PVP fibrous mats, the amorphous state of CIP, and its enhanced solubility in acidic conditions. Figure 8C shows the NMT released from microparticles S2. Initially, in the artificial gastric juice with a pH of 2, the cumulative NMT release was 3.7%, likely due to incomplete encapsulation in the ES100 shell coating during coaxial electrospraying. Later, in simulated intestinal fluid with a pH of 7.0, NMT release occurred in a sustained manner. The pulsatile release of CIP and the sustained release of NMT from hybrids S3 are displayed in Figures 8D, E, respectively, with release curves similar to those in Figures 8B, C. This evidence suggests that hybrids S3 demonstrates a combined release profile of electrospun nanofibers S1 and electrosprayed microparticles S2.

The NMT release profiles from microparticles S2 and hybrids S3 were further analyzed by regressing their *in vitro* drug release data according to the Peppas equation (Peppas, 1985). The drug-controlled release mechanisms for S2 and S3 are presented in Figures 8F,G, respectively. Their equations, i.e., $Q_{2}=8.98t1.04\left(R=0.9644\right)$ for S2 and $Q_{3}=7.67t1.11\left(R=0.9658\right)$ for S3, have drug release exponents of 1.04 and 1.11, respectively. These values, being greater than the critical value of 0.9, indicate that the drug NMT release was controlled by the erosion mechanism. In other words, the sustained release of NMT was regulated by the gradual dissolution or erosion of the shell ES100 and the core ES100 matrix.

### 3.5 The antibacterial performances of the hybrids

Prostatitis is primarily caused by *E. coli* infection, but other pathogenic bacteria such as anaerobes, *Proteus*, *Pseudomonas aeruginosa*, *Enterococcus*, *Mycobacterium tuberculosis*, *Neisseria* gonorrhea, fungi, trichomonas, *mycoplasma*, and *chlamydia*, can also contribute to the disease. CIP hydrochloride is a broad-spectrum antibiotic capable of inhibiting the growth and reproduction of a variety of bacteria, including Gram-negative and Gram-positive bacteria and anaerobes. Consequently, in this study, *E. coli* and *Staphylococcus aureus* were chosen as models of Gram-negative and Gram-positive bacteria to assess the antibacterial effects of electrospun nanofibers S1, microparticles S2, and hybrids S3.

Three time points (0.5, 4, and 8 h) were predetermined to perform the bacterial count and antibacterial rate experiments in the culture medium. All results are included in Table 2. The raw NMT powders exhibited a certain antibacterial effect. The NMT-loaded ES100 microparticles S2 also demonstrated antibacterial performance. As the incubation time increased, the antibacterial performance progressively improved, indicating the sustained release of NMT active ingredients. For *E. coli dh5α* and *Bacillus subtilis Wb800*, the increases ranged from 33.3% to 85.2% and from 31.6% to 84.1% after 0.5 and 8 h, respectively.

**TABLE 2:** The antibacterial results against *Escherichia coli* dh5α and *Bacillus subtilis Wb800* (*n* = 6).

Bacteria	Samples	Initial CFU	CFU after 0.5 h	CFU after 4 h	CFU after 8 h
			CFU (ABE%)	CFU (ABE%)	CFU (ABE%)
*Escherichia coli dh5α*	NMT	1.5 × 10^5^	6.7 × 10^4^ (62.8%)	7.8 × 10^4^ (88.4%)	2.2 × 10^5^ (89.5%)
	S1	1.5 × 10^5^	1.3 × 10^3^ (99.3%)	5.7 × 10^2^ (>99.9%)	4.1 × 10^2^ (>99.9%)
	S2	1.5 × 10^5^	1.2 × 10^5^ (33.3%)	1.2 × 10^5^ (82.1%)	3.1 × 10^5^ (85.2%)
	S3	1.5 × 10^5^	8.4 × 10^2^ (99.5%)	4.2 × 10^2^ (>99.9%)	1.4 × 10^2^ (>99.9%)
	Blank	1.5 × 10^5^	1.8 × 10^5^	6.7 × 10^5^	2.1 × 10^6^
*Bacillus subtilis Wb800*	NMT	1.5 × 10^5^	5.3 × 10^4^ (72.1%)	1.3 × 10^5^ (84.0%)	3.1 × 10^5^ (90.9%)
	S1	1.5 × 10^5^	7.7 × 10^2^ (99.6%)	4.1 × 10^2^ (>99.9%)	3.5 × 10^2^ (>99.9%)
	S2	1.5 × 10^5^	1.3 × 10^5^ (31.6%)	1.5 × 10^5^ (81.5%)	5.4 × 10^5^ (84.1%)
	S3	1.5 × 10^5^	5.6 × 10^2^ (99.7%)	2.8 × 10^2^ (>99.9%)	2.4 × 10^2^ (>99.9%)
	Blank	1.5 × 10^5^	1.9 × 10^5^	8.1 × 10^5^	3.4 × 10^6^

^a^ Abbreviations: NMT, Ningmitai powders (10 mg, an equal amount loaded in microparticles S2); CFU, colony-forming units; ABE, antibacterial efficacy.

CIP has robust sterilizing performance. The electrospun nanofibers S1 and fibrous sections in the hybrids S3 were able to release CIP molecules instantaneously. Therefore, it is unsurprising that the ABE reached values larger than 99% after 0.5 h of incubation. As a combination of nanofibers S1 and microparticles S2, the hybrids S3 demonstrated both a rapid initiation of antibacterial effect and a sustained antibacterial performance due to the continued release of NMT. Furthermore, NMT powders, which have several active ingredients and additional functions for treating prostatitis, demonstrate antibacterial properties that are relatively weaker than their other functional performances, such as clearing heat, detoxifying, promoting diuresis, and relieving gonorrhea. Hence, the co-loading of CIP in the EHDA hybrids S3 significantly improves the therapeutic effect of NMT for curing prostatitis. Systematic animal experiments and clinical trials will be further conducted.

### 3.6 Perspectives of the combined EHDA processes for structural nanomedicines

The focus of nanomedicine should be on exploring the new properties of medical materials that emerge when structures are manipulated at the molecular level (Muldoon et al., 2023). The future of nanomedicines increasingly relies on the development of novel structures and the associated fabrication techniques for potential clinical applications and commercial products.

Electrospinning has shown potential in advancing nanomedicine, particularly through the creation of multichamber nanofibers using multiple-fluid electrospinning processes. However, electrospraying and its multichamber structures have received comparatively less attention. This study pioneers the combination of electrospinning and electrospraying for fabrication. It combines nanofibers and two-chamber core-shell microparticles, soluble and pH-sensitive polymers for controlling separate sequential release profiles of multiple active pharmaceutical ingredients, and a traditional Chinese herbal medicine (NMT) with a traditional western medicine (CIP) for synergistic therapeutic effects.

**FIGURE 9:**
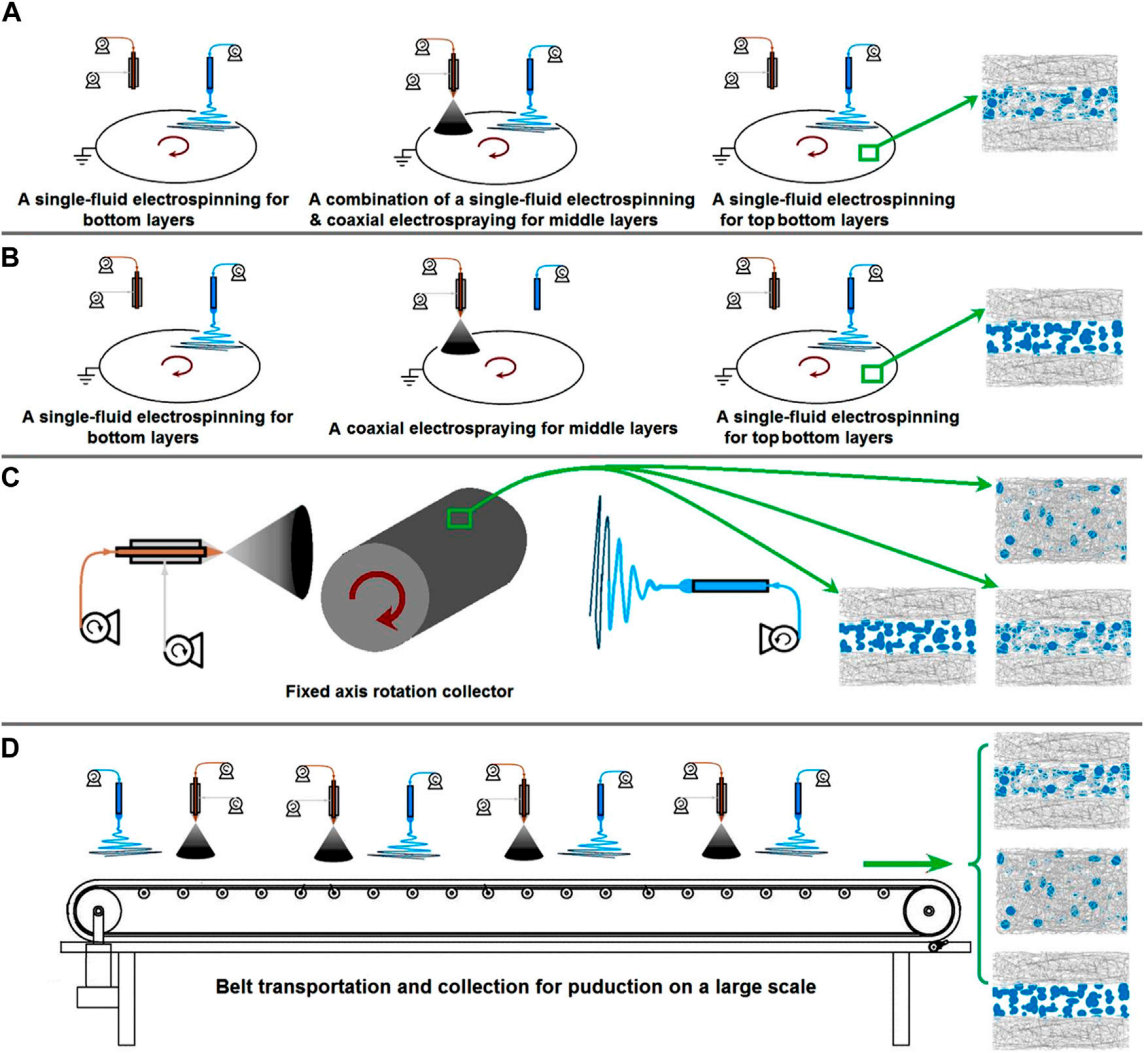
Perspectives of future studies: **(A)** the preparation of tri-layer fibrous mats through depositions of nanofibers/beads-on-a-string fibers/nanofibers; **(B)** the preparation of tri-layer fibrous mats through depositions of nanofibers/beads/nanofibers; **(C)** a diagram showing the preparation of several kinds of structural films through a fixed axis rotation collector; **(D)** the potential production on a large scale of multiple-layer fibrous products using belt transportation and collection.

Although the results indicate that the combination of various EHDA techniques is powerful for tailoring components, compositions, and organizational formats to further the development of novel nanomedicines, scaling up the production of EHDA nanomedicines and addressing related issues, such as energy conservation, toxic solvent usage, and safety measures, remain substantial challenges for researchers in the field (Kang et al., 2020; Brimo et al., 2022; Yu and Zhao, 2022). Several examples are illustrated in Figure 9. In Figure 9A, a procedure for preparing tri-layer fibrous mats via successive depositions of nanofibers, beads-on-a-string fibers, and nanofibers is displayed. Similarly, Figure 9B shows a procedure for preparing another type of tri-layer fibrous mat via successive depositions of nanofibers, electrosprayed beads, and nanofibers. The manipulation of EHDA product internal structures, external organizational formats, and the combination of electrospun nanofibers and electrosprayed particles would significantly enrich the design and fabrication of nanomedicine-based products.

Certainly, there are no limitations to improving the EHDA apparatus. As an example, replacing the current axial-fixed rotating plate collector with a similar axial rotation collector (Figure 9C) can allow for the separate and simultaneous collection of different EHDA products, a feature commonly seen in many reports (Sivan et al., 2022a; 2022b; Chen K. et al., 2023; Zhou et al., 2023d). In addition, the traditional belt transportation method can be integrated into the EHDA process for potential large-scale production, as indicated in Figure 9D. Above the collection and transportation belt, multiple needles for electrospinning and electrospraying can be arranged in parallel for the designed fabrications.

In this study, only CIP and NMT were encapsulated into the EHDA hybrids S3 to test the concept of combining coaxial electrospraying and electrospinning for fabricating hybrids that can sequentially release drugs to treat bacterial prostatitis. Future studies may consider loading other additives into the hybrids. For example, the blood-prostate barrier, a non-static physical barrier between the prostate stroma and the lumen of the prostate gland tube, strictly controls the mass exchange between the blood and the prostate, limiting drug penetration into the prostate (Naber et al., 1993; Liu et al., 2021). Therefore, pharmaceutical excipients that enhance drug penetration across the blood-prostate barrier could be loaded into the shell coating of ES100 to improve drug delivery. By the way, the fate of drug molecules is influenced by many factors (Wu and Li, 2022; Dong et al., 2023; Man et al., 2023; Zhang et al., 2023), the *in vivo*/*in vitro* drug delivery relationships and the final clinic results would be the most useful demonstrations for the applications of new methods of creating medicated materials, which will be further investigated.

## 4 Conclusion

In this study, we demonstrated a proof-of-concept that a combination of a Chinese medicine and a Western medicine could be used to treat prostatitis, each with its own accurately controlled release profile. We developed a combined EHDA process, which involves coaxial electrospraying and traditional single-fluid blending electrospinning, to create a new type of micro/nano hybrids for encapsulating these two medicines. The resultant hybrids comprise electrospun PVP-CIP nanofibers and electrosprayed core-shell microparticles, as verified by SEM and TEM assessments. XRD and FTIR experiments indicated that the drugs were present in an amorphous state with excellent compatibility.

Several in-house experiments demonstrated that the Western drug CIP, loaded into the electrospun nanofibers, could be released in a pulsatile manner upon exposure to water. *In vitro* dissolution tests verified that the hybrids were capable of providing colon-targeted sustained release of the Chinese medicine NMT, which was manipulated by the pH-sensitive polymer ES100 and a blank coating on the drug-ES100 composites. Furthermore, *in vitro* antibacterial experiments showed that the hybrids had superior performance to both the electrospun nanofibers and the electrosprayed microparticles in terms of quick antibacterial action and prolonged antibacterial effect over a relatively extended time period.

## Data Availability

The original contributions presented in the study are included in the article/supplementary material, further inquiries can be directed to the corresponding authors.
